# Application of the Numerical Model to Design the Geometry of a Unit Tool in the Innovative RTH Hydroforming Technology

**DOI:** 10.3390/ma13235427

**Published:** 2020-11-28

**Authors:** Hanna Sadłowska, Andrzej Kochański, Magdalena Czapla

**Affiliations:** Faculty of Production Engineering, Warsaw University of Technology, 00-661 Warszawa, Poland; akochans@wip.pw.edu.pl (A.K.); magdalena.czapla.stud@pw.edu.pl (M.C.)

**Keywords:** rapid tube hydroforming, rapid manufacturing, granular materials modelling, numerical modelling, Mohr–Coulomb hypothesis

## Abstract

The article presents a newly patented rapid tube hydroforming (RTH) manufacturing method, perfectly suited to single-piece production. The RTH technology significantly complements the scope of hydroforming processes. Due to the unusual granular material of the die tool, in particular moulding sand or mass, the process design requires the use of numerical modelling calculations. This is related to the complexity and the synergistic effect of process parameters on the final shape of the product. The work presents the results of numerical modelling studies of the process, including the behaviour of the die material and the material of the hydroformed profile. The numerical calculations were performed for a wide range of parameters, and can be used in various applications. The significant properties of moulding material used for the RTH tests were determined and one was chosen to build the die in RTH experiments. The results of the numerical modelling were compared with the results of the experiments, which proved their high compatibility. The final conclusions of the analyses indicate that the RTH technology has many possibilities that are worth further development and research.

## 1. Introduction

Tube hydroforming is a relatively young metal forming technology, and the first applications date to the 1940s. The first patents [1,2] concerning this technology revealed that the technology could be aimed at producing thin wall components with complex shapes and demanding applications. The constant development of hydroforming technology is due to the clear advantages of this forming method over other cold forming methods, among which the following benefits should be mentioned:

- more complex shapes in fewer operations;
- lighter components with the same strength or stiffness [3].

These features, combined with the use of advanced materials and new measurement methods such as [4], make hydroforming applicable in many industries, e.g., aerospace, automotive, agricultural machinery, and medical equipment [5,6,7,8,9,10].

**Figure 1:**
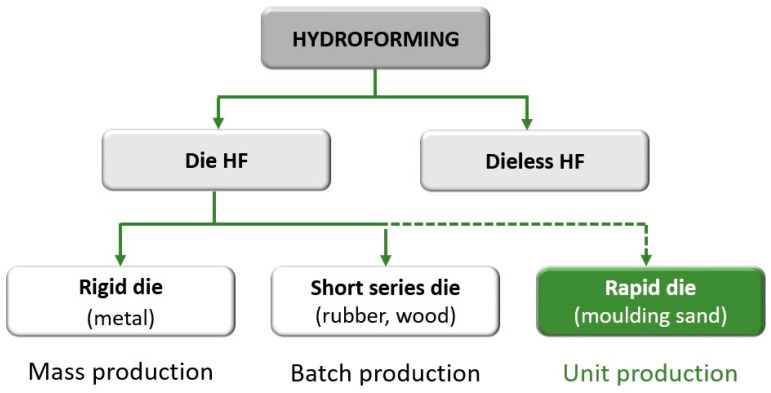
New classification of hydroforming technology [13,14].

The low manufacturing cost is another feature of hydroforming that makes it attractive for the forming of hard-to-work materials. However, profile forming in the hydroforming process so far has been limited by the time and cost of producing dies, hence meaning that low unit costs could be achievable only in long series. The economically reasonable amount for classic hydroforming (for rigid dies) was 1000 pieces of the product. The introduction of plastic, concrete or hardwood dies has made production profitable in batches of several hundred. However, this is still not cost effective to produce details in short series or prototypes. Today’s market requires a quick and individualized approach to the expectations of the industry machines and devices. This means that the production of prototype devices, single or very short series, will probably increase in the near future. The authors of the study set out to develop a hydroforming method suitable for short series, or even piece production. As hydroforming machines are universal and allow for the forming of profiles in a wide range of dimensions, the implementation of this goal is necessary to develop a method of quick and cheap production of dies. The authors proposed a complete change in the approach to the role and behavior of the die during the hydroforming process. In typical hydroforming, the die does not undergo plastic deformation leading to its destruction. In the new approach, a one-time, cheap matrix is completely destroyed, but this destruction is fully controlled. In order to control the die deformation process, advanced numerical modelling methods had to be incorporated into the technology design process. The recent development of such methods has allowed the use of numerical modelling in many aspects of the analysis of the shaping process, even as complex as, for example, forecasting the formation of defects [11]. The use of numerical modelling, in the case of this proprietary method, allows the development of a new method of hydroforming individual parts, without limiting the materials used. The rapid tube hydroforming (RTH) method, recently patented by the authors [12], makes hydroforming cost-efficient for single-piece production. The new method has thereby complemented the range of hydroforming technology and allowed for its use in the full range of production volumes, as represented in Figure 1 which highlights the new completed classification of hydroforming methods [13,14].

**Figure 2:**
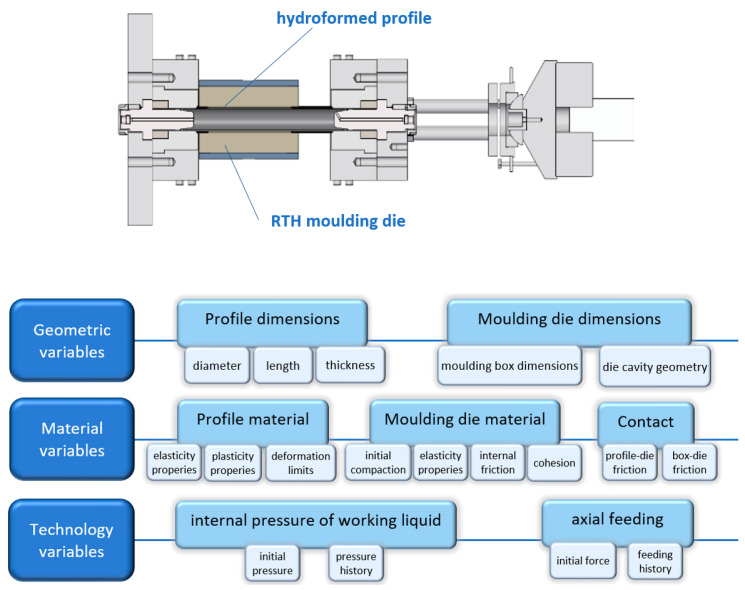
Rapid tube hydroforming (RTH) process scheme and its variables.

In regular hydroforming processes, as in many other metal forming techniques, the die cavity is designed to force the final product shape. That means that the hydroformed profile is the only one which undergoes plastic deformation and the die remains rigid. Moving away from this principle allowed for the use of other susceptible materials on dies, being much cheaper and easier to obtain. In this way, the idea of using granular materials for dies in hydroforming technology, e.g., moulding sand or mass, came up. This means that both the product and the tool, are being shaped during forming operation, in accordance with their respective material properties. This required the development of a method to control the die deformation process during profile deformation. According to the patent claims, the hydroformed profile deforms the die cavity and this acts in a fully controlled and intended way during hydroforming. The deformation of the profile is therefore the result of the current state of deformation of the die at the contact area. The process of deformation of the die during profile forming is based on the scenario predicted in the numerical simulation. The high complexity of the RTH method is related to several process variables: geometric, material, and process parameters, as schematically shown in Figure 2.

To fully control the profile forming process in the RTH method, it is important to know how the moulding mass die is deformed. This appears to be crucial when it comes to the proper design of the entire RTH process and, first, the original shape of the die cavity. There are a few points to consider when analysing this issue. The key question is what properties of moulding sand affect the deformability of the die cavity, and then, what initial values they would take to successfully shape the profile using the RTH method. Only the determination of the above enables one to make a final choice of material for the designed RTH process.

The studies in this article have identified the material parameters of moulding masses used in RTH matrices and investigated the possibility of forming profiles. The essence of the studies presented in the article is to demonstrate the effect of the properties of the masses on the deformability of RTH matrices, since this issue is necessary for the full control of the RTH process. The article also shows the results of an experiment confirming the correctness of numerical modelling of the process.

## 2. RTH Die Parameters

The essence of the new technology is that the die cavity material properties are more crucial in contrast to the classic metal or hydroforming processes. The most important assumption for classic tools is that its strength significantly exceeds the limit of plastic deformation of the shaped material. The die is supposed to deform in the elastic range of strain and has contact impact on the shaped material. In the new RTH technology, the condition of greater tool strength is not met, but also strength properties change during the forming process. Another novelty is the fact that the properties of the die vary in different zones and result from the forming element current state. This is the result of the behaviour of die material, which can thicken and move as a result of the push of the deformed element. Both of these phenomena lead to local changes in die properties.

The complexity of the RTH process, due to the multitude of variables affecting the final shape of the formed profile, presupposes the use of numerical tools to support the process design. It is necessary to define the appropriate parameters of the die, which include its initial shape, as well as the properties before and during the deformation process.

Tools made of moulding sands are not new, but their previous use in foundry processes as a sand mould, where excessive deformation is a defect, does not cover the needs of the RTH method. Therefore, the study analysed the behaviour of the moulding mass during profile formation, taking into consideration the knowledge gained from soil mechanics [15]. This approach allows to determine the deformation of the die, taking into account the mechanical properties of the test mass. The deformability of moulding mass as a granular material is associated with many properties such as compressibility or compactness, which are related to the physical parameters of the granular medium. However, from the point of view of the RTH process design analysis, one of the most important features of a fragmented centre is its strength, i.e., the load response. The strength of the granular medium is measured by the value of the shear stress resulting from the normal forces acting on the specimen. The boundary criterion describing the phenomenon of destruction in soils is explained in the Mohr–Coulomb (abbreviated as M–C) hypothesis, which unites the limit shear stresses to normal stresses. According to this hypothesis the medium strength depends on the cohesion c and the angle of internal friction φ of the granular material. Both parameters determine the internal resistance of the material related to shear stress caused by normal load [16].

Previous studies on phenomena associated with the compaction of granular material, including moulding masses, have indicated [17,18] that the M–C criterion can be successfully used for numerical modelling and analysis of mass movement and compaction under the influence of external forces. This approach prompted the authors to focus on the internal friction angle φ and the cohesion c as substantial factors of moulding sands used in RTH processes.

### 2.1. Material for RTH Die

Initially, it was necessary to specify the range of the examined values of c and φ before significance analysis of die behaviour in RTH processes. Therefore, an overview of the various materials belonging to the group of granular media and soils was conducted, choosing materials from loose sands to hard rocks [19]. A wide range of studies and accurate values of the cohesion coefficient and the angle of internal friction can be found in the soil and geomechanics literature, including studies on soil—which is a mixture of sands and clays [20]—and even on topics as exotic as soils on other planets [21].

In addition, the literature describing the mould sand’s properties [17] was used in determining the c and φ range. Forming masses used in foundries have rarely and a very small extent been studied in this respect and the literature on the subject gives approximate or wide ranges [17,19,22]. The methods have developed over the years and are commonly used in foundries to measure mass fluidity, mass density or mass properties correctly to determine the ability of a particular mass to produce foundry forms.

On this basis, the width of the cohesion coefficient c and the internal friction angle φ which are the critical values for the M–C model, are indicated; c = 0.1–5.0 MPa, and φ = 10°–50° [14]. The lower values were assigned to the moulding masses, and the upper values were assigned to materials belonging to fragmented centres in the field of geology, i.e., soil and rocks. The adoption of such a wide range of variability of both parameters requires confirmation in practical studies that an actual moulding mass can obtain such a range of variability.

### 2.2. Determining the Properties of Moulding Sand

**Figure 3:**
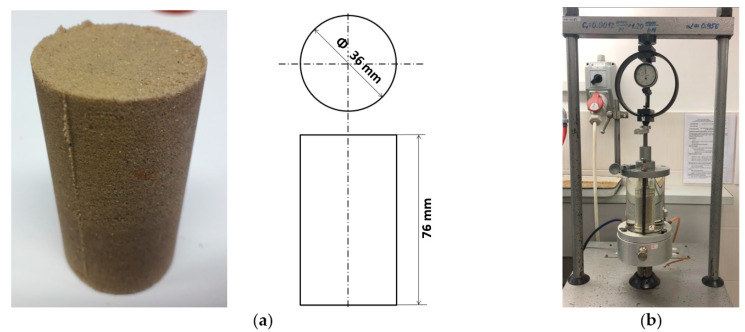
Sample test made of synthetic bentonite mass (**a**) and triaxial test site (**b**).

The cohesion coefficient c and the angle of internal friction φ of granular materials may be examined in the triaxial shear test described, e.g., in standard test [23]. The test is performed on standard cylindrical samples with a diameter of Ø36 mm and a height of 76 mm, as shown in Figure 3.

The known moulding sands were selected for preliminary studies: synthetic bentonite mass and water glass mass. The experiments aimed to determine the possible range of variability of the analysed parameters in moulding masses. In the latter case, an unusual content of ingredients was used. The test samples were made from mould sand Grudzeń Las 0.32/0.40/0.20. The compositions of both masses are given in Table 1.

**Figure 4:**
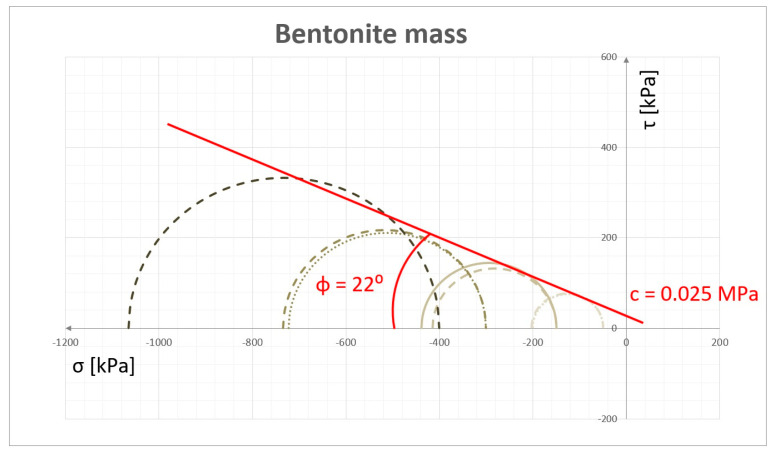
Examples of cohesion coefficient c and inner friction angle φ for bentonite mass.

Measurements of the shear strength of the moulding mass were made using the AT-3 three-axis compression device, see Figure 3b. The experiments for both materials were performed at different values of hydrostatic pressure (50, 150, 200, 300 and 400 kPa) and at an axial force until the sample cracked. As a consequence of the grain structure of examined materials, the triaxial tests are characterized by a high degree of uncertainty of the results, which is due, e.g., to the mixing method and its compaction. To improve the results, at least three measurements were made for each pressure. Examples of test results, in the form of M–C wheels, are shown in Figure 4. The cohesion coefficient c, specified for the test mass, was 0.025 MPa and the internal friction angle φ was 22°.

According to the methodology of the triaxial test, the internal friction coefficient is defined as the angle of inclination of the line tangent to the M–C wheels, while the value of the cohesive coefficient c is determined as the intersection of this tangent with the Y axis.

**Figure 5:**
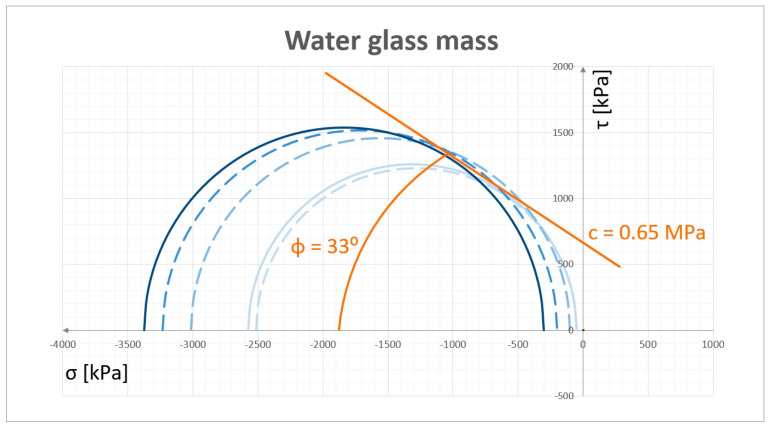
Examples of cohesion coefficient c and inner friction angle φ for water glass mass.

For the mass with water glass the corresponding test and analysis were performed, and sample results are shown in Figure 5. The cohesion coefficient c specified for the test mass was 0.65 MPa and the internal friction angle φ was 33°.

It is worth realizing that the investigated parameters are in the range of known physical properties of the moulding mass. The investigated materials are a good example including a strong relationship between the internal friction with the size and shape of the grain warp [24]. This can be confirmed by results of the performed tests and the fact that the moulding sands have a wide-ranging grain size, coefficient of grain shape and surface character. It makes obtaining moulding masses with pre-established parameters possible, i.e., a cohesion coefficient c in the range of 0.1–1.5 MPa and an angle of internal friction φ in the range of 10°–55°.

## 3. Numerical Analysis of RTH Dies

To estimate the significance of the internal friction angle φ and the cohesion coefficient c in the RTH process, a numerical model based on the Mohr–Coulomb hypothesis described earlier was developed. It allowed numerical experiments to be made taking into account the wide range of the two parameters tested. Numerical calculations were implemented to examine the effect of c and φ parameters on mass deformability under a given load.

**Figure 6:**
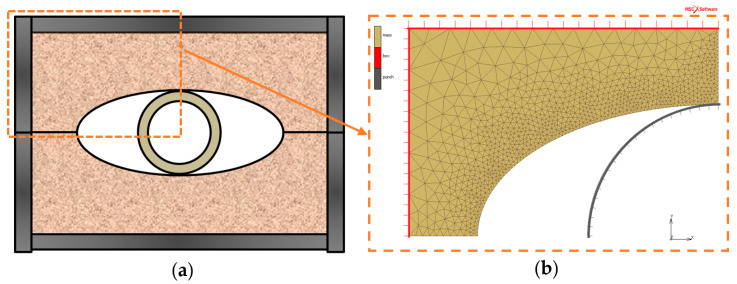
RTH numerical model: (**a**) scheme of the real RTH process, (**b**) numerical model geometry.

When developing a numerical model to analyse RTH die behaviour during tube hydroforming, the basic die geometry with an ellipse-shaped cavity was adopted. A cylindrical element (pretending to be a hydroformed tube) was placed inside the die and was deformed by increasing its diameter. This study intended to investigate the behaviour of dies made of different materials (with different combinations of c and φ) in a repeatable way of deformation of a cylindrical element. Therefore, the expanding element has been modelled as a rigid part that increases its diameter evenly and the effect of the deforming die on it has not been directly analysed. Thanks to this, the effect on die deformability could be analysed independently of the variable response of the real hydroformed tube. The analysed die was assumed to be 110 mm × 80 mm and the ellipsoidal cavity was assumed to be 72 mm × 38 mm. The expanding element (punch) had an initial Ø 38 mm diameter that ensured initial contact with the cavity from the beginning of the simulation. The deformation of the die continued until the punch reached a diameter of 48 mm. The geometry of the RTH process used in the numerical analysis and the corresponding computer model are shown in Figure 6.

Numerical calculations were performed using MSC.Marc software (ver. 2019, MacNeal-Schwendler Corporation (MSC), Newport Beach, CA, USA) that allowed to define the material model using the Mohr–Coulomb strength hypothesis. As mentioned above, the numerical model used to analyse the impact of parameters c and φ is based on the interaction of an expanding punch deforming the moulding die. The plain strain triangular elements in the number of 1745 were used to mesh moulding die material. The number of elements and grid arrangement were determined during the model calibration process taking into account the criterion of calculation stability and the calculation time minimization. An elastic–plastic model with a plastic criterion based on the Mohr–Coulomb linear model was used to describe the behaviour of the moulding mass [25]. The elastic deformation part is defined by Young modulus E = 100 MPa and the Poisson coefficient ν = 0.2 (according to [26]). Calculations were made by entering the Coulomb coefficient of friction between the die and the punch as a value of 0.1.

**Figure 7:**
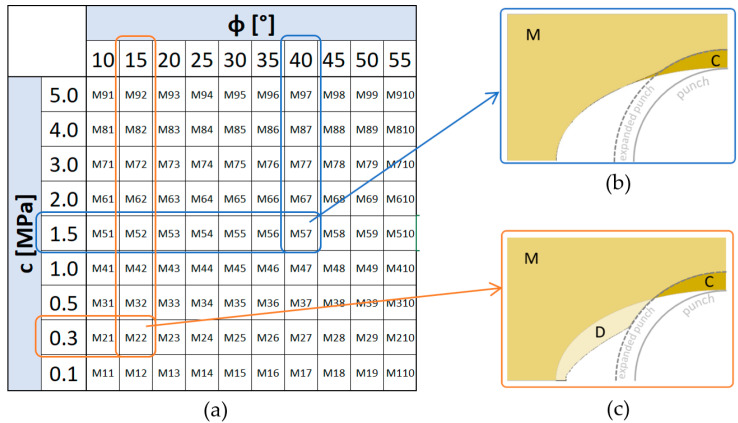
Numerical experiments of RTH: (**a**) case table, (**b**) RTH die deformation for high internal resistance, (**c**) RTH die deformation for low internal resistance.

There were a number of calculations performed for a range of data that were determined as described in Chapter 2, i.e., for the internal friction range φ from 10° to 55° and for cohesive c from 0.1 to 5 MPa. All analysed cases are shown in the table in Figure 7a.

When analysing the results, the main focus was on the deformation of the die and the degree of its movement under the influence of the movement of the expanding punch. Since the tested c and φ parameters represent the internal resistance of the die material, it was expected that for high values of both parameters the mass would deform mainly around the contact location with the punch. This could indicate the smaller impact of the punch on the other regions of the die. On the other hand, for masses with lower internal resistance, i.e., for low values c and φ, the mass would not only undergo deformation in contact with the punch, but would also move to further regions of the die. Numerical calculations confirmed this hypothesis and thus made it possible to identify parameters favourable to the appearance of these two characteristic extreme occurrences of mass deformation: with deformation in the punch area and with additional mass pushed into further fragments of the die. Examples of the two different moulding mass behaviours are shown in Figure 7b,c. Figure 7b shows the final deformation stage of the RTH die for a mass with relatively high internal strength, characterized by the properties c = 1.5 MPa and φ = 40° (calculation case M57, Figure 7a). It can be observed that the punch pushes the mass just close to the contact area (area C). The mass in the unsupported area of the die (area M) does not experience significant transposition and deformation. The extremely different nature of the moulding mass behaviour can be illustrated in Figure 7c, which represents the deformation of the RTH die made of material with much less internal strength (c = 0.3 MPa and φ = 15°, computational case M22, Figure 7a). It can be observed, in addition to moving region C deep into the die, that the mass also moves into the unsupported space between the die cavity and the punch (area D). This ease of movement occurs in accordance with the phenomenon of lower internal mass resistance, i.e., the smaller cohesion and the angle of internal friction. Some designations of characteristic regions were introduced. Thus, area C can be defined as the one in which the mass tends to be compacted, while area D is part of the mass which can be easily displaced and in which the density is unlikely to occur (dilated).

**Figure 8:**
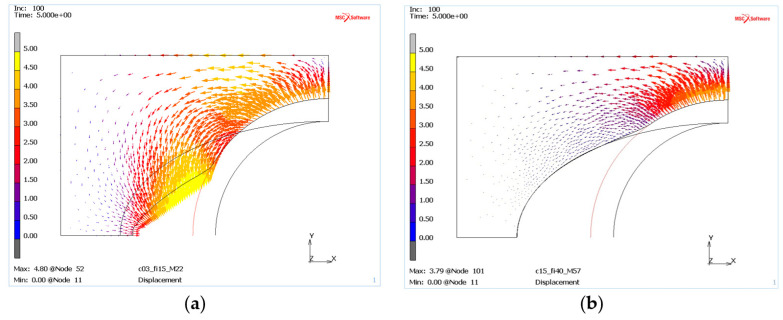
The mass displacement vectors as result of punch movement for two different die materials: (**a**) c = 0.3 MPa and φ = 15°, (**b**) c = 1.5 MPa and φ = 40°.

The simulations also allowed us to study the nature of mass movement in the deformed die, which can be easily observed from the distribution of displacement vectors, see Figure 8. Calculations for “weak” die material (Figure 8a) and the “resistant” material (Figure 8b) with displacement vector distributions openly show significant qualitative differences, indicating a lack of material movement in the zone of punch contact. There can be also quantitative variance, i.e., the length of displacement vector, which is starting to increase for a low c and φ mass and is up to 4 mm, Figure 8a.

**Figure 9:**
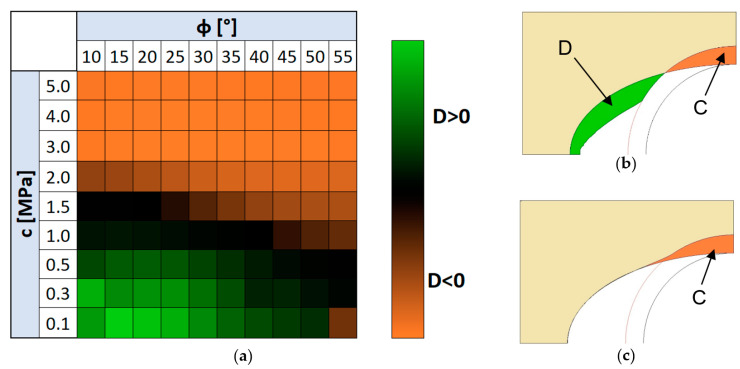
Summary of RTH die behaviour for different materials: table of all investigated cases (**a**), case for masse with low cohesion (**b**), case for mass with high cohesion (**c**). Legend: green: the mass moves into the space between the punch and the die (D > 0), orange: the mass does not travel into the space between the punch and the die (D ≤ 0).

The results shown in Figure 8 regarding the characteristic die behaviours were so interesting that other computational cases were analysed in this respect. To make this easier, a division criterion was introduced which distinguished between cases where the mass not only suffered displacement due to direct stamp pressure (area C), but also moved below the original die cavity (area D > 0), see Figure 9b. These cases are shown in green in the table in Figure 9a, other cases, that is, those in which the mass is moved only above the cavity profile (area C) are indicated in orange. The analysis of Figure 9 shows that the effect of the frictional angle on the behaviour of the mass is relatively small, and at the same time it is not difficult to see that with the stamp forcing, the moulding mass with a cohesion of more than 1.5 MPa practically does not move into the free space between the die cavity and the punch (D ≤ 0), see case in Figure 9c. This means that RTH dies made of masses with high internal pressure based on cohesiveness will tend to behave similarly to rigid matrices, of course, when it comes to the nature of the reaction due to the deforming profile rather than the magnitude of the contact pressures. Low-pressure masses will be much more susceptible to deformation, and their shape and deformation will also be able to affect the deformed profile outside the primary contact region.

The applied Mohr–Coulomb material model in MSC.Marc does not allow for direct determination of mass density, but by analysing the strains in the X and Y directions it is possible to conclude where the mass tends to be compacted or where it is free to move without any compaction inclination. The tendency for compaction can be linked to negative strains in both the X and Y direction. On the other hand, the positive strains indicate mass regions with unrestricted displacement of the die material.

**Figure 10:**
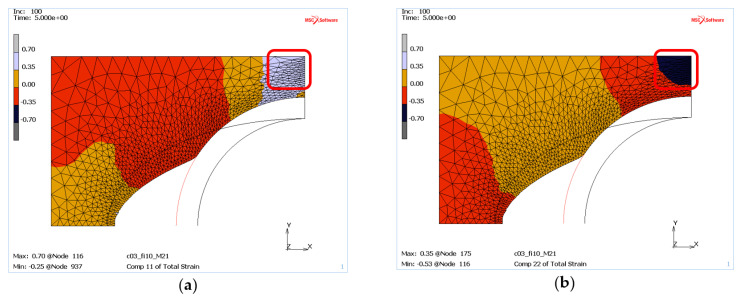
The die deformation for a mass with low internal pressure (case M21): (**a**) deformation on the *X*-axis, (**b**) deformation on the *Y*-axis.

Thus, for the “weak” mass (Figure 10) located near the contact zone with the punch, the material is pushed in the Y direction, as evidenced by the negative strain values, which can indicate its compaction (Figure 10b). However, at the same time positive strains on the X-axis (Figure 10a) are observed, suggesting the movement of the mass in the direction of the free space between the punch and the die. The described locations are marked with red boxes in Figure 10.

**Figure 11:**
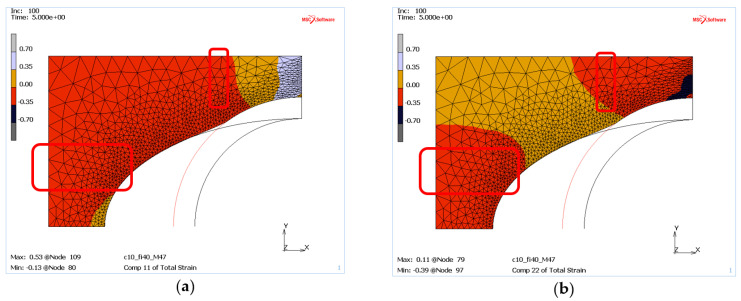
Deformations of die material with high internal pressure (case M21): (**a**) deformation on the *X*-axis, (**b**) deformation on the *Y*-axis.

On the other hand, for masses with higher internal resistance, e.g., c = 1.5 MPa and φ = 40°, areas of compaction tendency can be certainly observed where the deformations in both directions are negative, see red frames in Figure 11.

**Figure 12:**
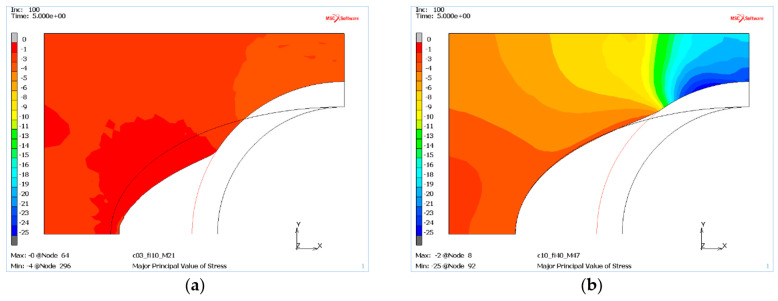
The major stress for a deformable die of two different masses: (**a**) with low internal pressure (case M22), (**b**) with high internal pressure (case M47).

The numerical analysis of the RTH die for a wide range of parameters has highlighted an additional, important aspect for the entire RTH profile forming process. The stress distribution caused by the moving punch has revealed a mass response for the established deformation. It can be seen that the stresses are several times higher for a mass with high c and φ values than for a weaker mass, see Figure 12 showing the major stress distribution for the two types of die materials. This can result in the profile being more ellipsoidal.

To conclude the numerical analysis, it can be observed that for different mass parameters it is possible to obtain a high shape variation of RTH dies, which, in combination with the deformation-induced die material response, can be essential for the deformability of the hydroformed profile in the real RTH process. In addition, for the analysed die shape and the assumed deformation, mass deformations have been shown to be significant only for limited narrower ranges c and φ. This is particularly obvious in the case of cohesion, where increasing above 1.5 MPa does not produce visible die deformation. Thus, dies made of high cohesion materials have a much higher chance of effectively supporting the shaped profile in the actual RTH process.

## 4. RTH Experiments

**Figure 13:**
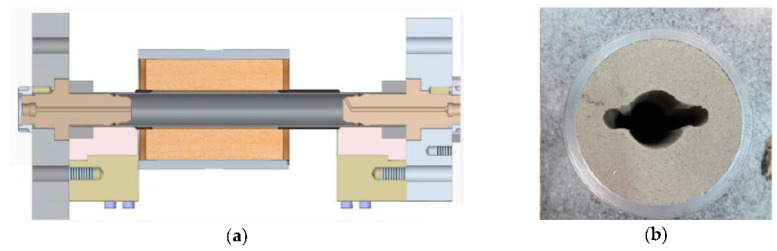
The RTH experiment on TH research stand: (**a**) scheme and tools, (**b**) die cavity profile [12].

To verify the usefulness of the Mohr–Coulomb model to describe the behaviour of the moulding die in computer simulations, a new numerical model of thin-walled steel profile hydroformed in the moulding die has been built. The results obtained using this model were compared to the results obtained in the real RTH experiment. For this comparison, a particular test was developed at the TH (Tube Hydroforming) research stand [11]. The experiment involved a E235 + N steel tube (Ø38 × 2), hydroformed in a die made of water glass mass with the physical properties described in Chapter 2. In Figure 13 the scheme of the RTH experiment and the die cavity geometry are shown.

**Figure 14:**
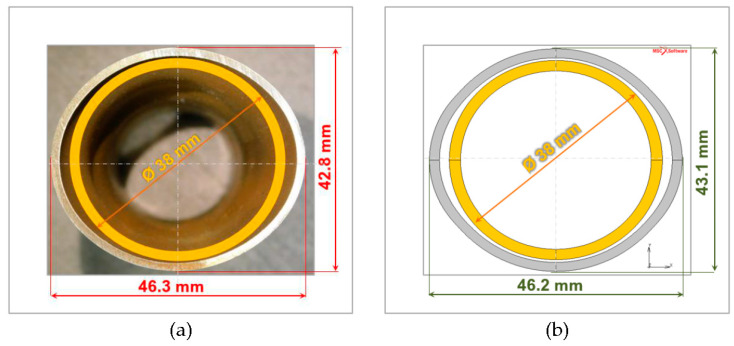
The comparison of experiment (**a**) and numerical (**b**) result of RTH process.

For both profile and die geometry, the information used in the experiments was entered into the numerical model. The die was modelled according to the Mohr–Coulomb criterion using the material data for the water glass mass described in Chapter 2. For the steel profile an elastic–plastic model with Huber-von Mises-Hencky’s criterion was adopted. According to the RTH method the tube was formed by the internal pressure of the working liquid, changing as in the experiments. The performed RTH tests clearly demonstrated the ability of the moulding die to sustain the deforming profile, which was assessed by roundness deviation analysis. For the selected experimental cases cross-sections of the initial and final outlines were measured and the results were compared with the equivalent dimensions obtained in the numerical simulation. In one of the experiments, a circular tube with an initial diameter of 38 mm was hydroformed and finally acquired, with the shape of an ellipse with minor and major axes of 42.8 mm and 46.3 mm, respectively. This is shown in Figure 14, which represents an experimental RTH case with a high compatibility of results being achieved.

**Figure 15:**
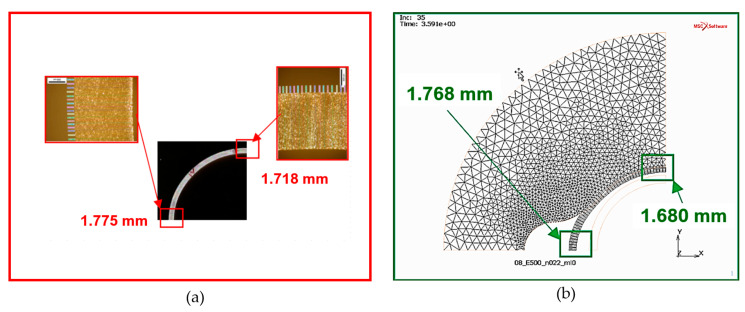
The comparison of the thickness of the characteristic regions of the hydroformed tube: (**a**) the results obtained in experiments, (**b**) the numerical simulation results.

A critical limitation in hydroforming, is the acceptable thinning of the profile wall depending on its material and geometry and how it is deformed. Due to the unusual association between the die and the profile, it was important to take a closer look at the behaviour of the cross-section profile and to analyse the thickness distribution. The microscopic examination of the thickness distribution was performed for selected sections of the deformable profile. The results of the measurements of two critical section points are shown in Figure 15. As can be observed, this aspect also achieved high compatibility between modelling and experimentation.

## 5. Conclusions

The presented research entirely confirmed the usefulness of the new patented RTH method for the hydroforming of single parts or short series. The existing hydroforming methods using die tools made of inexpensive materials, such as hard wood or plastic, for economic reasons were limited to the production of more than several dozen pieces. The new RTH method has repealed this limitation. Unlike hydroforming in plastic or wood dies, the method allows to form profiles at elevated temperatures. Due to the use of materials known in foundry, the method has no limitations on the height of the profile forming temperature.

The numerical modelling of the RTH process showed a significant influence of the properties of the moulding sand on the behaviour of the die during the forming process. The wide variability of the die deformation character justifies the conclusion that there is a possibility of using the RTH method in a wide range of materials and geometry of the profiles. Numerical analysis of the RTH process showed the important effect of moulding mass parameters on the deformability of the RTH die. These two properties (internal friction angle and cohesion coefficient) have been shown to be key parameters influencing the determination of the shape of the initial die, which will guarantee that the final shape of the hydroformed profile will be as designed.

Numerical analysis also showed that high cohesion masses deform with much greater difficulty and thus generate significant resistance to the eventually deformed thin-walled profile process.

The experiments verified the numerical modelling at two levels, i.e., the profile shape obtained and the section geometry (wall thickness). In both cases, the results of modelling and the actual experiment showed a far-reaching compatibility with the resulting profile shape, demonstrating the high effectiveness of the developed hydroforming method.

The new method of tube forming, due to its individual character, seems to be excellent for application, and also for materials with unique properties, e.g., high-pressure steels or magnesium alloys. Further research is necessary to determine the material properties of the profile to be shaped and the interaction between the mass properties of the die and the properties of the material.

## Figures and Tables

**Table 1:** Chemical composition of tested masses.

Bentonite Mass		Water Glass Mass	
Sand:GrudzeńLas 0.32/0.40/0.20	88.6%	Sand:GrudzeńLas 0.32/0.40/0.20	92.6%
Bentonite	8.9%	Sodium water glass R-145	6.5%
Water	2.6%	Ester hardener (of ethylene glycol diacetate)FlodurFM-4	0.9%

## References

[1] Squires J. (1936). Method of Making Airplane Propeller Blades. U.S. Patent.

[2] Matsukin J. (1964). Method for Hydrodynamic Forming of Bellows-Type Articles and a Device for Their Realization. U.S. Patent.

[3] Bell C., Corney J., Zuelli N., Savings D. (2020). A state of the art review of hydroforming technology: Its applications, research areas, history, and future in manufacturing. Int. J. Mater. Form..

[4] Sadłowska H., Morawiński Ł., Jasiński C. (2020). Strain measurements in free tube hydroforming process. Arch. Metall. Mater..

[5] Kocańda A., Sadłowska H. (2008). Automotive component development by means of hydroforming. Arch. Civ. Mech. Eng..

[6] Han S., Woo Y., Hwang T., Oh I., Moon Y.H. (2019). Tailor layered tube hydroforming for fabricating tubular parts with dissimilar thickness. Int. J. Mach. Tools Manuf..

[7] Wagner S.W., Ng K., Emblom W.J., Camelio J.A. (2017). Influence of Continuous Direct Current on the Microtube Hydroforming Process. ASME J. Manuf. Sci. Eng..

[8] Reddy P.V., Reddy B.V., Ramulu P.J. (2020). An investigation on tube hydroforming process considering the effect of frictional coefficient and corner radius. Adv. Mater. Process. Technol..

[9] Saboori M., Gholipour J., Champliaud H., Wanjara P., Gakwaya A., Savoie J. (2016). Prediction of Burst Pressure in Multistage Tube Hydroforming of Aerospace Alloys. ASME J. Eng. Gas Turbines Power.

[10] Hyrcza-Michalska M. (2017). Research on liquid forming process of nickel superalloys thin sheet metals. Arch. Metall. Mater..

[11] Cai G., Fu J., Zhang D., Yang J., Yuan Y., Lang L., Alexandrov S. (2020). A Novel Approach to Predict Wrinkling of Aluminum Alloy During Warm/Hot Sheet Hydroforming Based on an Improved Yoshida Buckling Test. Materials.

[12] Kochański A., Sadłowska H. (2020). Sposób Hydromechanicznego Kształtowania Profili Cienkościennych i Matryca do Hydromechanicznego Kształtowania Profili Cienkościennych. Patent.

[13] Kochański A., Sadłowska H. (2020). Rapid Tube Hydroforming—The Innovative Casting-Forming Method for Rapid Prototyping. Manuf. Technol..

[14] Kochański A., Sadłowska H. (2020). A Casting Mould for Rapid Tube Hydroforming Prototyping. J. Cast. Mater. Eng..

[15] Dmitruk S., Izbicki R., Suchnicka H. (1982). Mechanika Ośrodków Ciągłych.

[16] Murthy V.N.S. (2002). Geotechnical Engineering: Principles and Practices of Soil Mechanics and Foundation Engineering.

[17] Waszkiewicz S. (1992). Wpływ Błędów Odwzorowania Wnęki Formy na Dokładność Wymiarową Odlewów.

[18] Bast J., Malaschkin A., Kadauw A., Betschweia I. (2007). Formstoffcharakterisierung durch Simulation des Verdichtungsprozesses. Giesserei.

[19] Dodge Woodson R. (2011). Appendix III—Foundry Sand Facts for Civil Engineers. Concrete Portable Handbook.

[20] Zydroń T., Dąbrowska J. (2012). The influence of moisture content on shear strength of cohesive soils from the landslide area around Gorlice AGH. J. Min. Geoengin..

[21] Sullivan R., Anderson R., Biesiadecki J., Bond T., Stewart H. (2011). Cohesions, friction angles, and other physical properties of Martian regolith from Mars Exploration Rover wheel trenches and wheel scuffs. J. Geophys. Res..

[22] Lewandowski L. (1991). Masy Formierskie i Rdzeniowe.

[23] ASTM International (2020). ASTM D4767-11. Standard Test Method for Consolidated Undrained Triaxial Compression Test for Cohesive Soils.

[24] Vangla P., Latha G.M. (2015). Influence of Particle Size on the Friction and Interfacial Shear Strength of Sands of Similar Morphology. Int. J. Geosynth. Ground Eng..

[25] MSC Marc 2019—Volume A: Theory and User Information. https://simcompanion.mscsoftware.com/infocenter/index?page=content&id=DOC9245.

[26] Patel A., Ingale R., Bhanarkar K.B. (2018). Effect of Compaction States and the Confining Pressure on Poisson’s Ratio of Stratified and Non-Stratified Soils. Arab. J. Sci. Eng..

